# Interdisciplinary interactive blended learning concept in times of a pandemic – pain medicine “totally digital”

**DOI:** 10.3205/zma001792

**Published:** 2025-12-22

**Authors:** Lisa Schramm, Patrick Friedrich, Jürgen Schüttler, Björn Lütcke

**Affiliations:** 1 University Hospital Erlangen, Department of Anesthesiology, Erlangen, Germany

**Keywords:** COVID-19, digitalization, e-learning, blended learning, pain medicine

## Abstract

**Introduction:**

Pain medicine is located in different sections of the medical curriculum. In the pandemic situation, an online teaching concept for Q14 which includes several disciplines had to be developed. The goal of the project was to create a fully digitized learning platform for the cross-sectional area Q14 that allows all participating disciplines to address the various learning goals without losing a practical component.

**Project description:**

First, the students' expectations regarding education in the field of pain medicine were recorded by means of a survey among medical students. Based on this, a teaching module in a blended learning format was developed, which consisted of two parts. Within a digital learning platform, students were first required to complete consecutive learning units using an interactive learning management system. This was followed by a presence phase (online ZOOM seminar) in which, under the guidance of teaching staff, the therapy suggestions of the individual case studies from the previous learning program were reflected. In the second part, the acquired knowledge was applied to a simulated patient. An evaluation of the online module was carried out through free-text answers and self-assessment of the completion time. The ZOOM seminar was evaluated on the basis of an assessment by the teachers.

**Results:**

The survey among students revealed a desire for practical training without “frontal teaching”. The resulting project realized this aspect by teaching theory during an online module with case vignettes and interactive learning tasks. The subsequent online presence time during the ZOOM meeting enabled the students to repeat and deepen contents and to ask questions. 170 students completed the entire online program, of which evaluation data were available for 75 students. Self-assessment of completion time averaged at 4-6 hours. In the feedback, 90 aspects were addressed, including mainly comments on content (43%), praise (33%) and comments on technical problems (23%). According to the assessment of the presenters, the students were able to carry out the pain anamnesis survey in a structured manner. The submission of the therapy proposal, however, represents a particular hurdle.

**Conclusion:**

With the presented blended learning concept it is possible to address the different learning goals and the interdisciplinarity of Q14 sufficiently. After further processing and improvement of the project, a controlled and more extensive collection of evaluation data is required to further investigate the benefit of the platform for the students regarding achievement of defined learning goals.

## Zusammenfassung

**Einleitung:**

Schmerzmedizin ist an mehreren Stellen des medizinischen Curriculums verortet. In der Pandemiesituation musste ein Online-Lehrkonzept für Q14 erarbeitet werden, das mehrere Fachdisziplinen einschließt. Ziel des Projektes war es, eine vollständig digitalisierte Lernplattform für den Querschnittsbereich Q14 zu schaffen, die allen beteiligten Disziplinen die Adressierung der verschiedenen Lernziele ermöglicht, ohne eine praktische Komponente zu verlieren.

**Projektbeschreibung:**

Zunächst wurden die Erwartungen der Studierenden an die Lehre im Bereich Schmerzmedizin mittels einer Umfrage unter Medizinstudierenden erfasst. Auf Grundlage dessen wurde ein Lehrmodul im Blended-Learning-Format erarbeitet, das aus zwei Anteilen bestand. Innerhalb einer digitalen Lernplattform wurden durch die Studierenden zunächst aufeinander aufbauende Lerneinheiten über ein interaktives Lern-Management-System verpflichtend absolviert. Es folgte eine Präsenzphase (Online-Zoom-Seminar) in der, unter Anleitung durch Lehrpersonal, zunächst die Therapievorschläge der einzelnen Fallbeispiele aus dem vorangegangenen Lernprogramm reflektiert wurden. Im zweiten Teil wurde das erarbeitete Wissen an einem Schauspielpatienten angewendet. Eine Evaluation des Online-Moduls erfolgte via Freitext-Antworten und Selbsteinschätzung der Bearbeitungsdauer. Das ZOOM-Seminar wurde Anhand einer Einschätzung der Lehrenden evaluiert.

**Ergebnisse:**

Die Umfrage unter Studierenden ergab den Wunsch nach praxisnaher Ausbildung ohne „Frontalunterricht“. Das entstandene Projekt realisierte diesen Aspekt durch die Theorie-Vermittlung während eines Online-Moduls mit Fallvignetten und interaktiven Lernaufgaben. Die anschließende Online-Präsenzzeit während des ZOOM-Meetings ermöglichte es den Studierenden, die gelernten Inhalte zu wiederholen und zu vertiefen und individuelle Fragen zu stellen. 170 Studierende absolvierten das gesamte Online-Programm, davon lagen für 75 Studierende Evaluationsdaten vor. Die Selbsteinschätzung der Bearbeitungsdauer lag im Durchschnitt bei 4–6 Stunden. Im freien Feedback wurden 90 Aspekte angesprochen, darunter vor allem inhaltliche Anmerkungen (43%), Lob (33%) und Anmerkungen zu technischen Problemen (23%). Nach Einschätzung der Moderatoren können die Studierenden in der Präsenzphase die Schmerzanamneseerhebung strukturiert durchführen, allerdings stellt die Unterbreitung des Therapievorschlages eine besondere Hürde dar.

**Schlussfolgerung:**

Mit dem vorgestellten Blended-Learning-Konzept ist es möglich die verschiedenen Lehrbereiche und die Interdisziplinarität von Q14 suffizient zu adressieren. Nach weiterer Bearbeitung und Verbesserung des Projektes ist eine kontrollierte und umfangreichere Erhebung von Evaluationsdaten nötig, um den Nutzen der Plattform für die Studierenden im Hinblick auf das Erreichen von definierten Lernzielen weiter zu untersuchen.

## 1. Introduction

**Figure 1:**
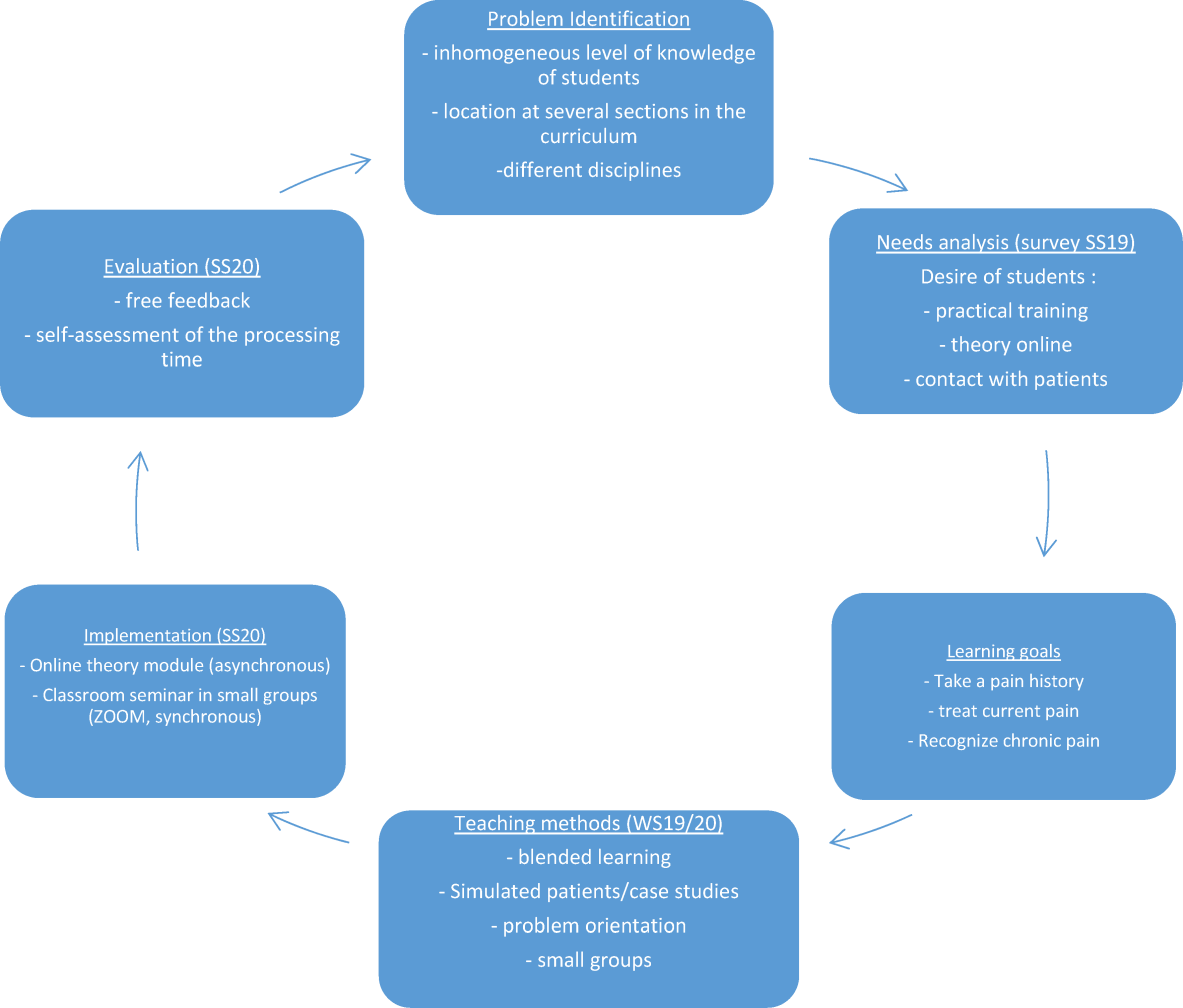
Development of the project based on the Kerns‘ cycle [4]

Pain medicine is located in several sections of the medical curriculum. Learning goals regarding Pain Medicine can be found in many disciplines and are summarized in Q 14. Within the cross-sectional area, higher-level learning objectives need to be addressed, which are defined in the National Competence-Based Learning Objectives Catalog of Medicine (NKLM). These include various competences such as identifying pain, applying or assigning treatment methods, and identifying main symptoms [http://www.nklm.de], [1], [2]. Different education concepts of the disciplines involved in Q14 (anesthesiology, psychosomatics, neurology, pediatrics, palliative medicine) pose a challenge for teaching. The prior knowledge of students in the field of pain medicine is equally heterogeneous. With this in mind, we have developed a blended learning concept in fall 2019, in which the needs of the students and the participating disciplines are equally addressed (“SchmerzAktiv 2.0”, see figure 1 (Fig. 1)). Due to the pandemic situation in spring 2020 and the necessity of digitizing the courses, the further development of the project took up an unforeseen speed. The concept was implemented through two consecutive teaching components. Students first completed an e-learning module and then participated in a face-to-face seminar, which was performed as an online group seminar due to the COVID-19 pandemic. The goal of the project was to create a fully digitized learning platform for the cross-sectional area Q14 that would allow all participating disciplines to address the various sub-areas of Q14 without losing a practical component.

## 2. Project description

The development of the entire teaching module was based on the evidence-based system for the development of medical curricula according to Kern [3] (see figure 1 (Fig. 1)). 

In a needs analysis, the expectations of students regarding education in the field of pain medicine were first recorded in the summer semester of 2019 (see figure 1 (Fig. 1)). This was done by means of a survey among 60 students in the 9th semester at the medical faculty of FAU Erlangen which was carried out by student assistants. Through an open question (“What wishes and expectations do you have for teaching in the field of pain medicine?”), the students had the opportunity to freely express their ideas. In addition, the results of the semester evaluations of the Students Office were included. The results were then compared with the technical possibilities of the ILIAS learning platform. Based on the “Core Curriculum Pain Medicine for Teaching a Cross-Sectional Subject of Pain Medicine According to the New AO” of the German Pain Society e.V. [4], [5] and following the National Competence Based Learning Objective Catalog of Medicine (NKLM), three overarching learning objectives were defined in the next step as

1. the ability to take a structured pain history,
2. the ability to treat acute pain with basic algorithms, and
3. the ability to recognize risk factors for chronification of pain.

Based on this, in the winter semester 2019/2020, the creation of the teaching concept and the development of the required content took place, which was necessary for the release of the first version of the online module in the summer semester 2020. Based on the evaluation data which was collected during the semester, the entire teaching format was then further developed and modified (for the winter semester 20/21). The steps described are illustrated in figure 1 (Fig. 1).

**Figure 2:**
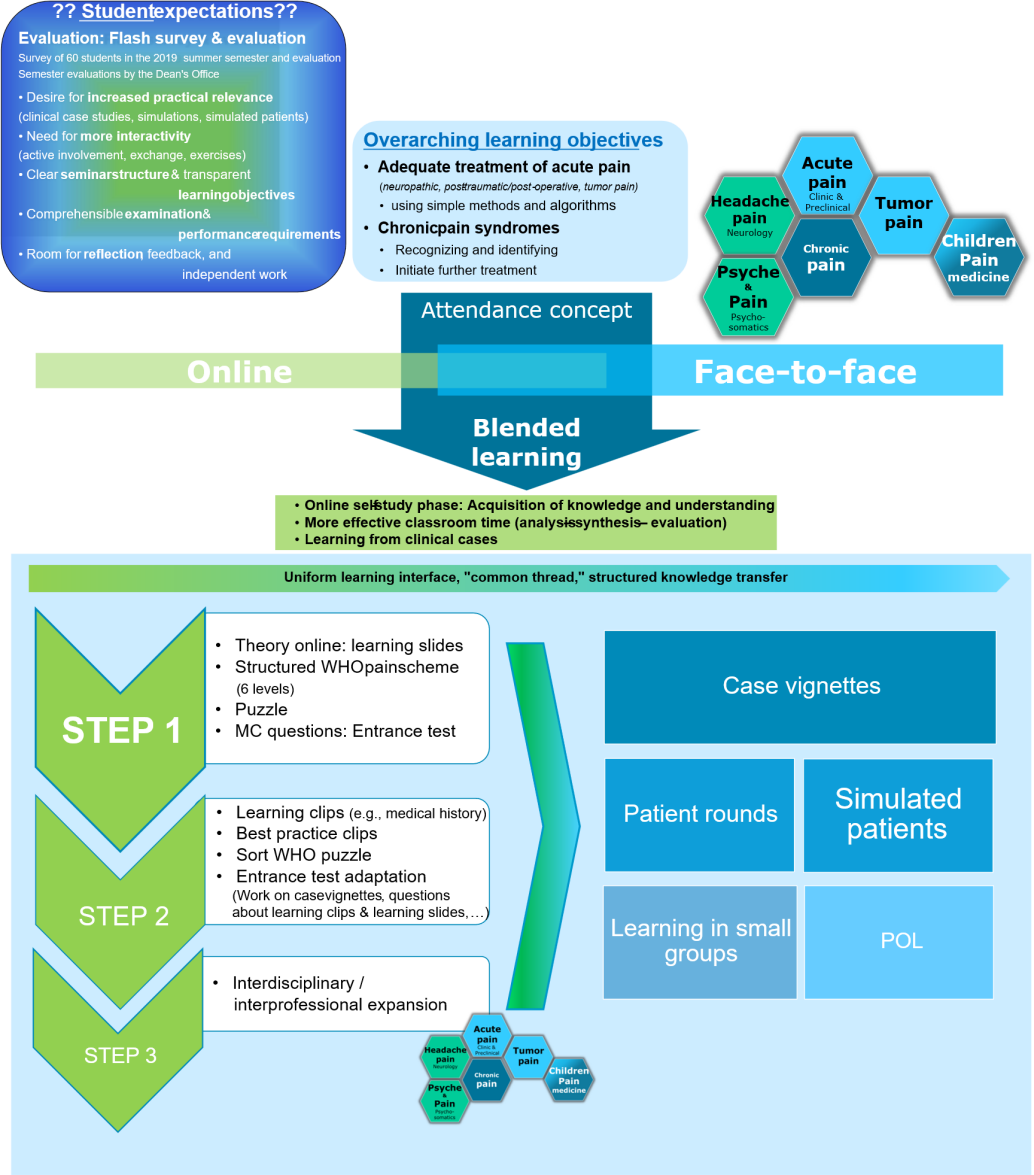
From classroom-based learning to blended learning: The blended learning concept is developed based on student expectations, learning objectives, and pain points. Development of online modules in three steps: STEP 1 – Theory and case vignettes, STEP 2 – Interactive videos, STEP 3 – Integration of other disciplines. In-depth classroom phase with presentation of own therapy proposals and interaction with simulated patients.

The implementation process was developed by staff members of the department of anesthesiology and was presented in a poster. After recording the challenges of education in the field of pain medicine and the ideas of the students as well as the definition of the learning goals and the teaching concept, a transformation of the previous attendance model into a blended learning concept, was given (see figure 2 (Fig. 2), upper part). The cornerstone of the concept is problem-oriented learning (“POL”) in small groups. This happened primarily through case examples in form of case vignettes but also simulated patients (see figure 2 (Fig. 2), lower part on the right). The further development and implementation of the project was performed in several steps during the following semesters. The goal was to gradually incorporate further learning methods such as videos and interactive elements into the learning platform. As a final step, additional disciplines should be included (see figure 2 (Fig. 2), STEP 1-3). An overview of the development and the initially planned implementation phases of the entire project is shown in figure 2 (Fig. 2).

In the summer semester 2020, the first step, which involved the theoretical transfer of knowledge (learning slides), the development of case studies and interaction with simulation patients, was fully implemented (see figure 2 (Fig. 2), “STEP 1”). In the learning management system ILIAS (open source e-Learning), the students initially created learning units that were built on each other in a self-directed rotation model and blended learning concept [6], [7]. Access to the module was activated one week before the scheduled face-to-face seminar. The learning platform was then processed asynchronously. Thus, each student had the opportunity to work on the topic at his or her own pace and at self-determined times, possibly also with an interruption of the module. The learning content in the online module was interactive and presented thematically (see table 1 (Tab. 1)). They included, among others, learning slides, a WHO stage scheme puzzle, intermediate questions in MC format, and case vignettes of various pain patients with audio files and situation pictures based on these (see figure 2 (Fig. 2)). For the case studies, students should develop their own therapy suggestions, which will be presented and discussed in the subsequent attendance phase.

The subsequent attendance phase took place in a synchronous course as an approximately two-hour ZOOM seminar (3 teaching units) in groups of 20 students. The prerequisite was the completion of the preceding online part. The seminar was led by one of three physicians who are experienced in pain medicine and had previously been trained in the implementation and content of the seminar. At the beginning of the event, the therapy suggestions of the individual case studies from the online module were first reviewed together and then questions were specifically addressed. 

In the second part, the acquired knowledge was applied in a conversation with a simulation patient. The actor was familiarized with his role at the beginning of the semester and trained accordingly by colleagues experienced in pain medicine. Attention was paid to both verbal and nonverbal communication and certain gestures and facial expressions.

During the seminar, one student took on the role of a doctor. The task for this part was to take a structured pain history during an empathetic patient interview followed by presenting a therapy suggestion to the patient. The other students were given observation tasks (communication, doctor-patient relationship, therapy suggestions, medical aspects, etc.). After completion of the patient interview, these aspects were compiled and discussed in the group.

In the planning of 2019, the concept was initially designed for acute pain therapy and the field of anesthesiology. However, due to the COVID-19 pandemic, it became necessary to include other disciplines at an early stage of project development. Thus, in the summer semester of 2020, it was possible to also involve the specialties of neurology and psychosomatics. 

In this project phase, the evaluation took place after completion of the online module and was integrated. Sociodemographic data and a self-assessment of the completion time were collected. In addition, students had the opportunity to provide open feedback through free text comments. These comments were reviewed and categorized. Comments that were addressed multiple times by students were presented as examples. Results per category were given as absolute and relative frequencies. The presence phase (Online-ZOOM-Seminar) was evaluated on the basis of an assessment by the teaching staff in a personal interview.

## 3. Results

In the survey conducted among students, the desire for case-based, practice-oriented training emerged. Theoretical content should be taught online to avoid so-called “frontal teaching”. In addition, the students wanted teaching with contact to patients (see figure 1 (Fig. 1) “Flash”). Attendance time should be used more effectively for practical training, but also for clarifying questions. These results suggested a blended learning concept in which online theory teaching and presence phases are combined [7], [8], [9]. Practical relevance and case-based work were realized through several patient cases in the online phase as well as through training on an simulated patient during the presence phase, enabling deepening of knowledge. Through the mandatory completion of the preceding online part, a homogenous horizon of expectations was set for the students. In the following attendance phase this served as a tool for active and effective participation in the seminar. It also represents a guideline for the design of the meeting by the teachers. Based on practical case studies with a simulated patient, a structured pain anamnesis was taken within the group seminars, thus repeating the main contents. This concept allows the students both, an active training on the patient example and the individual consideration of questions and ambiguities. Due to the COVID-19 pandemic and the resulting restrictions on attendance phases in student teaching, the group seminar was conducted entirely online as a ZOOM meeting in the summer semester of 2020.

**Figure 3:**
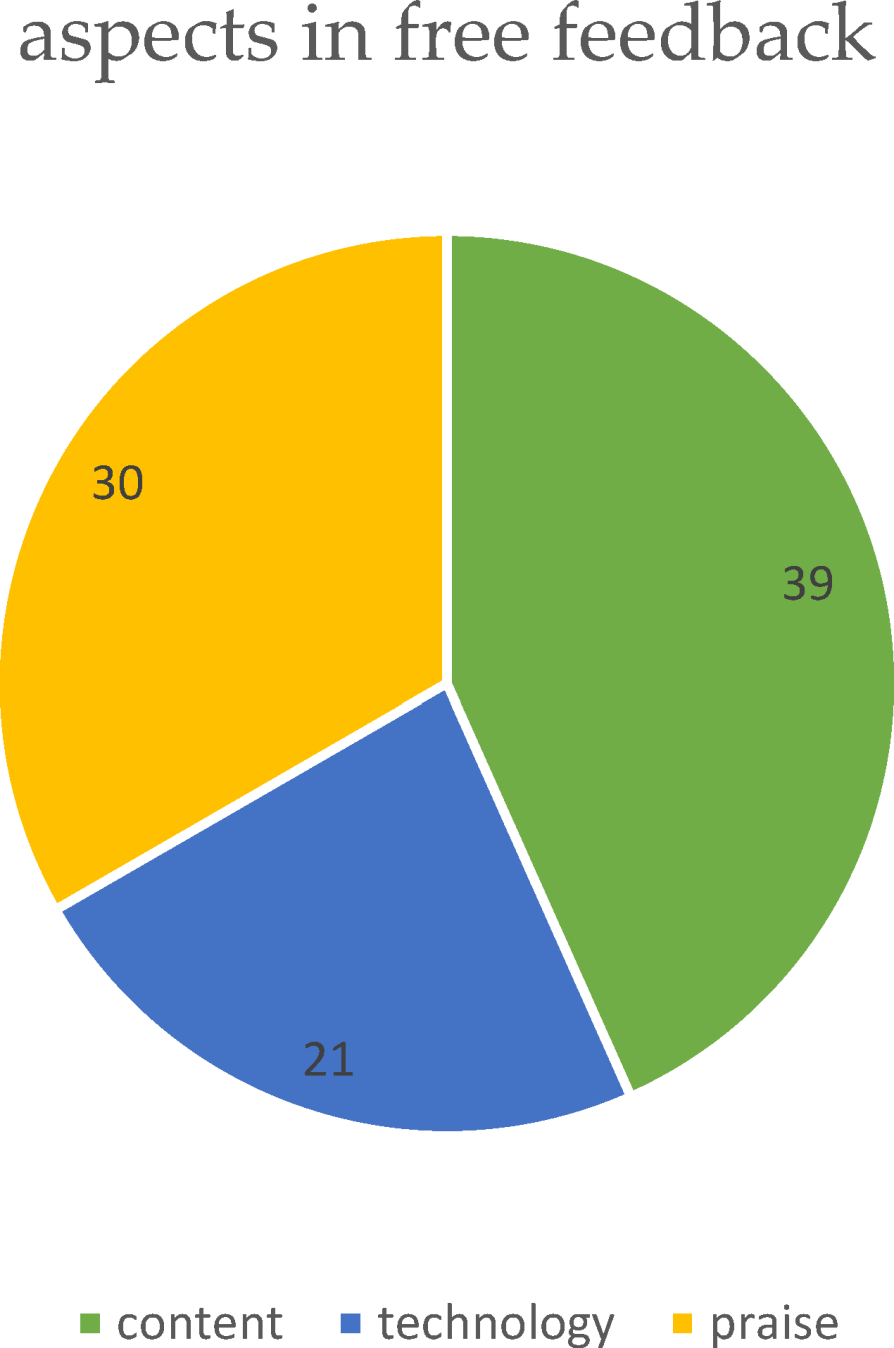
Aspects addressed by students in free feedback

170 students completed the online phase and the ZOOM presentation seminar. Of these, data was available for 75 students. The students were in the 9^th^-11^th^ semesters of study. 90% (n=68) reported German as their native language. The self-assessment of the processing time was on average 4-6 hours (approx. 8 teaching units). A total of 90 aspects were addressed in the free feedback. These included content-related questions and suggestions (n=39, 43%), technical problems (n=21, 23%) and praise (n=30, 33%). The results are shown in figure 3 (Fig. 3).

**Figure 4:**
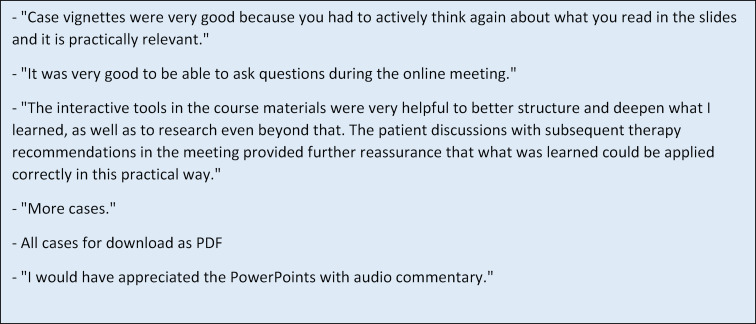
Feedback and optimization suggestions from participants

Aspects that were mentioned multiple times in the students' free text comments are exemplified in figure 4 (Fig. 4). 

According to the instructors' assessment, the students are able to carry out the pain anamnesis survey in a structured manner in the ZOOM presence phase. However, in their opinion, the subsequent submission of the therapy proposal represents a major challenge.

## 4. Discussion

Teaching pain medicine has several challenges. On the one hand, pain medicine is anchored at various points in the course of the medical curriculum. Second, multiple disciplines have commonalities with pain medicine content. This circumstance leads to a very fluctuating prior knowledge of the students and complicates a homogeneous and tangible education in this field, which is of great importance in everyday medical practice.

Schulz et al. encountered a similar problem when developing a teaching concept in the field of palliative medicine. They were able to show that digital teaching concepts, blended learning, and interprofessional teaching formats offer a way to handle this complex specialty [10]. Ruiz and colleagues also see the combination of digital blended learning concepts and traditional teaching as a great opportunity for medical education [11]. Therefore, a blended learning concept proved to be a suitable format for redesigning teaching in the cross-sectional area 14. Through the additional use of simulation patients, students can practically apply the previously learned content. They are a good tool for realistically presenting case studies and various interview situations, as Schulz and colleagues also showed [12]. The pandemic situation necessitated collaboration between various specialist disciplines with different training concepts and focuses. The interdisciplinary communication and linking was noticeably accelerated by the existing time pressure, so that a uniform provision of content from all departments could be realized via a common online platform.

Based on the feedback of the students, a further elaboration of the learning slides (e.g. with dubbing and possibility for download) would be desirable in the online learning module. In addition, more different case studies need to be developed to make the learning platform even more varied. The presence phase was held, differently than originally planned, as a ZOOM video seminar. Here it was shown that students are able to conduct discussions with drama patients in a ZOOM meeting and subsequently also present therapy proposals. The submission of the therapy schemes is a particular hurdle. A best-practice example (e.g. as a video in the online phase) could ensure more security for the students in their role as a doctor. In addition, aspects of taking a medical history and developing therapeutic approaches could be illustrated more practically with an interactive video in H5P format (see figure 2 (Fig. 2), “STEP2”). This would give the students a better opportunity to grasp the different phases of pain medicine care. During the course of the semester, the lecturers repeatedly provided feedback regarding the time required for the seminars. The e-learning module was estimated to be approximately 8 teaching lessons (depending on the time taken by the students to complete it) and the face-to-face phase 3 teaching lessons. With regard to the face-to-face seminar, in the feedback from the teachers and the students it became clear that the estimated time was not sufficient in many cases to discuss all questions and comments in detail. Together with the further aspect that simulated patients are a good instrument to deepen the learned skills, an extension of the attendance phases to more teaching units in the next semester would be desirable. In this way, more simulated patients could be involved in different roles and more students would have the opportunity to slip into the medical position. In the future, it would also be desirable to integrate other departments, such as pediatrics and palliative medicine (see figure 2 (Fig. 2), “STEP 3”).

### Limitations of the study 

The entire development and implementation process of the described project required an enormous amount of human and financial resources. Especially in times of COVID-19, where every resource was needed in the daily clinical routine (in intensive care units, in the operating room, etc.), it was often difficult to manage the necessary time and personnel for the implementation of the project. The high commitment of our working group and the support (financial as well as personnel) by our department had great influence on the successful implementation of the project.

A central limitation of the project is the shortage of time in the implementation of the learning platform, which is why the data collection was not designed as a study concept. The primary goal of this project was first to investigate whether it is possible to design a complex and interdisciplinary subject like Q14 completely digitally without losing the practical component. As a result, the evaluation data has a purely descriptive character and can only depict the benefit of the platform for the students with regard to the achievement of learning goals to a limited extent. In addition to the points described above, controlled and more extensive data collection by means of evaluation should take place in the further course of the project in order to investigate the influence of the processing of the e-learning platform on the students' level of knowledge and the practical application of this.

## 5. Conclusion

The various sub-areas of pain medicine (Q14) can be represented very well in an online teaching concept (e-learning and online presence phase). Practical aspects can be presented in doctor-patient discussions (students - acting patients). Interdisciplinary aspects can be made visible through case-based learning both in the online platform and in the presence phase.

From our point of view, the results indicate that it is possible to depict the different sub-areas of Q14 adequately with a completely online teaching concept (e-learning and online presence phase) and to present the interdisciplinarity of pain medicine without losing the visibility of the participants.

## Acknowledgement

Many thanks to our colleagues at the department of anesthesiology and the pain outpatient clinic, who gave us professional advice in the selection and elaboration of the patient cases and the implementation of the project. Additional thanks goes to our student assistants C. Arntzen and R. Hotop, who actively supported us in the development and technical implementation of the project. Many thanks also to our colleague Dr. A. Schmidt from the Skillslab PERLE for her advice and proofreading of this article. Last but not least, many thanks to our clinic director Prof. Dr. Dr. J. Schüttler for the opportunity to implement this interesting and great project.

## Competing interests

The authors declare that they have no competing interests. 

## Note

This article is the corrected version of an article originally published in the journal in 2022 [13], [14].

## Figures and Tables

**Table 1:**
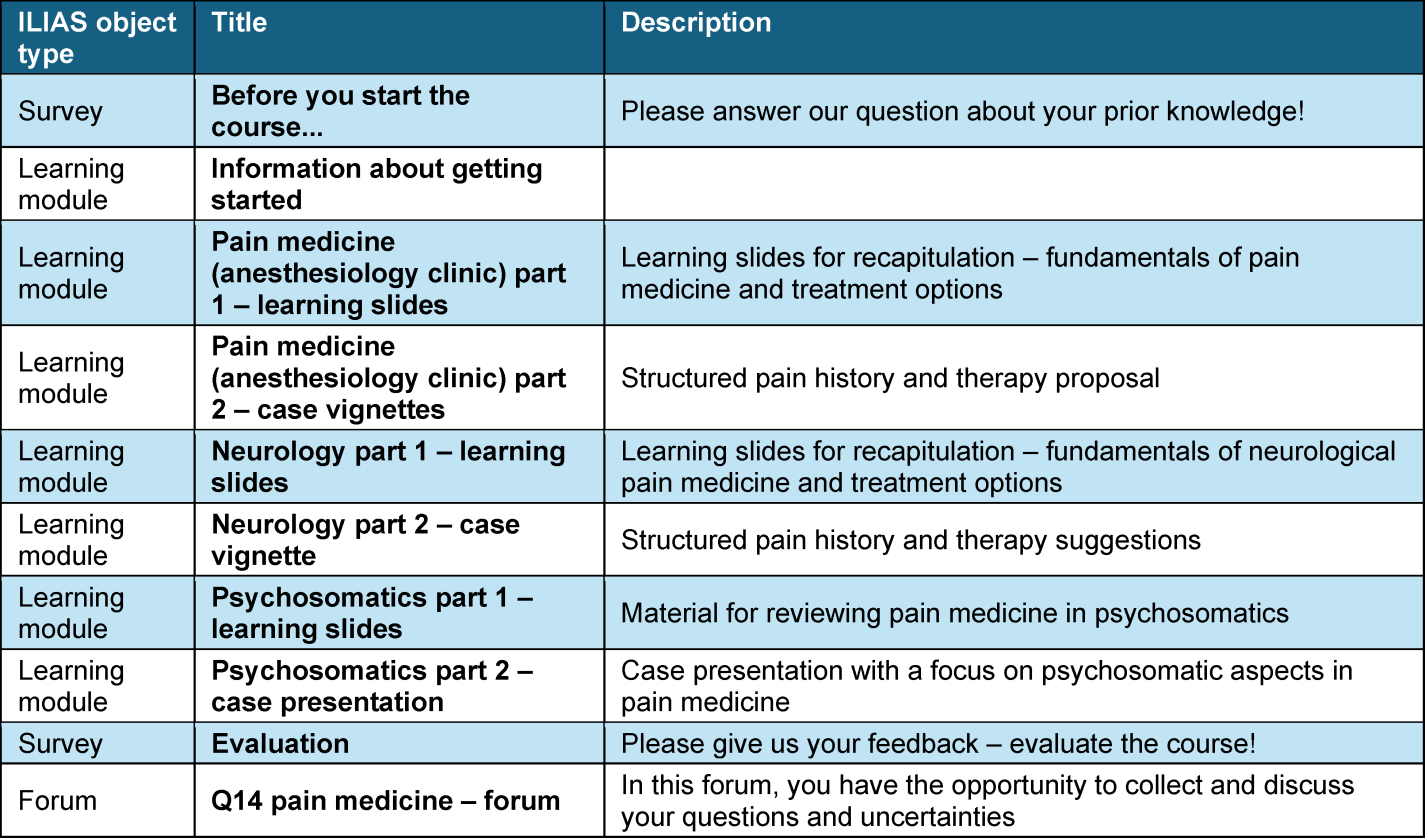
Overall content and sequence of the individual learning sections in the ILIAS learning management system
